# Effects of Three Modification Methods on the In Vitro Gastrointestinal Digestion and Colonic Fermentation of Dietary Fiber from Lotus Leaves

**DOI:** 10.3390/foods13233768

**Published:** 2024-11-24

**Authors:** Hui Zheng, Yao Xu, Yuhang Wu, Xuantong Huangfu, Wenxiu Chen, Kai He, Yong Yang

**Affiliations:** 1 College of Pharmacy, Hunan University of Chinese Medicine, Changsha 410208, China; huizheng0104@163.com (H.Z.); 20223684@stu.hnucm.edu.cn (Y.X.); 20233794@stu.hnucm.edu.cn (Y.W.); huangfu3431@163.com (X.H.); 19386957996@163.com (W.C.); 2 School of Pharmaceutical Science, Hunan University of Medicine, Huaihua 418000, China

**Keywords:** lotus leaves, dietary fiber, polyphenols, colonic fermentation, shear emulsifying, ball milling, autoclave treatment

## Abstract

Shear emulsifying (SE), ball milling (BM), and autoclave treatment (AT) were utilized for the modification of lotus leaves, and the effects on in vitro gastrointestinal digestion and colonic fermentation of insoluble dietary fiber (IDF) from lotus leaves were compared. Compared with SEIDF and ATIDF, BMIDF released more polyphenols and exhibited better antioxidant capacity during in vitro gastrointestinal digestion. The IDF of lotus leaves changed the gut microbiota composition during in vitro colonic fermentation. SEIDF was beneficial to the diversity of gut microbiota compared with BMIDF and ATIDF. Among the three IDF groups of lotus leaves, six significant differences of OTUs were all in ATIDF; however there was the highest relative abundance of Escherichia-Shigella in ATIDF. In addition, the concentrations of butyric acid and valeric acid produced by SEIDF were significantly higher than that of BMIDF and ATIDF. Overall, SE modification improved the colonic fermentation characteristics of IDFs in lotus leaves more effectively; while BM modification helped to promote the release of polyphenols from IDFs in lotus leaves during in vitro gastrointestinal digestion. The research lays the foundation for the application of the dietary fiber of lotus leaves as a premium fiber additive in functional food.

## 1. Introduction

Most of the dietary fiber (DF) discovered so far comes mainly from the cell walls of plants, which are resistant to digestion by the body’s own enzymes and therefore provide almost no calories [1,2]. In recent years, DF has emerged as an exceptional raw material for the production of functional foods that cater to various wellness needs, such as weight loss, blood lipid reduction, blood sugar management, and gastrointestinal regulation [3]. As the secondary metabolites of plants, polyphenols are abundant in many plants. During the preparation of natural plant DFs, it has been found that polyphenols may remain in DFs through chemical bonding or physical bonding with DFs. Some of these polyphenols can be released from DFs during digestion in the gastric and intestinal tract, where they can regulate insulin release and metabolize proteins in small intestinal epithelial cells [4], while others can reach the colon, where they exhibit their excellent antioxidant properties to eliminate free radicals and regulate gut microbiota [5,6]. Increasing the bioavailability of polyphenols has become a hotspot for research. It has been demonstrated that bioavailability of polyphenols was related to DF content, particle size, and environmental factors, such as pH, temperature, and ionic strength [5,7]. Zhao et al. found that ultrafine rice bran IDF powder exhibited increased polyphenol extractability, polyphenol bioaccessibility, and antioxidant activity compared to rice bran IDF coarse powder [8]. DFs can be categorized into insoluble dietary fiber (IDF) and soluble dietary fiber (SDF) based on their solubility in water. IDFs mainly include cellulose, hemicellulose, lignin, and resistant starch in plant cell walls. Studies have shown that many IDFs exhibit superior water-holding capacity, oil-holding capacity, and swelling property compared to SDFs [1]. The characteristics of IDFs facilitate gastrointestinal motility, lipid encapsulation, carbohydrate digestion, and heavy metals sequestration in the human body. However, IDFs are mostly dense and cross-linked structures with large crystalline regions, which makes them poorly soluble. Solubility has been shown to be closely related to intestinal fermentation capacity, most IDFs are less fermentable or non-fermentable in the colon [9]. Studies have shown that loosening the dense structure of IDFs or binding to antioxidant components can enhance their functional properties [10,11].

Lotus leaves are the leaves of the perennial aquatic plant Lotus (*Nelumbo*), which is widely grown and cultivated in both the north and south of China. Lotus leaves exhibit various beneficial effects, such as antibacterial, anti-inflammation, lipid-lowering, and antioxidant activities, and have been officially recognized by the Chinese Ministry of Health for both medicinal and food applications [12]. However, the dense fibrous structure of lotus leaves makes them difficult for direct utilization in food products. Currently, research on lotus leaves primarily focuses on alkaloids and polyphenols, with limited attention given to the fiber component [12]. In our previous study, we demonstrated that DF from the encapsulated polyphenolic compounds of lotus leaves was more beneficial for human health [13]. It is vital to apply suitable modification methods to loosen the structure and enhance the functional properties of IDFs from lotus leaves. By breaking the glycosidic bonds of carbohydrates, one can obtain IDF with diverse structures and change their properties [14]. Physical modifications commonly utilize high-temperature, high-pressure, high-speed impact, and shearing to break the glycosidic bond of DFs [15]. Ball milling (BM) utilizes thermal effects to assist high-speed collision forces in altering the properties of raw materials and has become a common fiber modification technology [16]. Autoclave treatment (AT) has been widely used in food processing and sterilization technology. AT employs high pressure and high heat treatment, which is capable of disrupting the dense structure of raw materials, and this disruption leads to alterations in the functional properties [17]. Shear emulsification (SE) uses the high-speed rotation of the rotor to produce a large fluid shear force for modifying the structure and performance of the raw material, and has been utilized in the dispersion, emulsification, homogenization, and crushing processes of food materials [18]. BM employs high-speed impact, AT utilizes high temperature and high pressure, and SE involves fluid shear, representing three distinct modification principles. This study uses SE, BM, and AT to modify the structure of IDFs in lotus leaves. By comparing the physicochemical properties and polyphenol compounds of the three modified IDFs in lotus leaves, it was found that SE modification was more beneficial for loosening the dense fiber structure of IDFs in lotus leaves, while BM modification was more beneficial for increasing the polyphenol binding capacity of lotus leaves IDF [13]. To further explore the effects of three modifications on the functional characteristics of IDFs in lotus leaves, this study compared polyphenol release and antioxidant activities of processed IDFs during in vitro simulated gastrointestinal digestion. Their effects on gut microbiota during in vitro fermentation were also investigated.

## 2. Materials and Methods

### 2.1. Materials

Dried lotus leaves were sourced from Zhenxing Medicine Co., Ltd. (Changsha, China). All chemicals were commercially available and classified as analytical grade.

### 2.2. Lotus Leaves Modification Treatment and IDF Preparation

The powder of dried lotus leaves was produced by grinding the leaves using a pulverizer and then passing it through a 50-mesh sieve. For SE modification, 25 g of lotus leaf powder was added to 750 mL of distilled water. This mixture was subjected to shearing at 8000 rpm for 30 min using the FM20-D shear emulsifier (Shanghai Fluko Technology Development Co., Ltd., Shanghai, China). The precipitate was collected after centrifugation at 5000 rpm for 15 min and subsequently freeze-dried to obtain SE modified lotus leaf powder. For BM modification, 50 g of powder was mixed with 0.9 kg of zirconia balls in a one-liter container. This mixture was then processed using the XQM-4 planetary ball mill (Changsha Tianchuang Powder Technology Co., Ltd., Changsha, China) at 500 rpm for 30 min to obtain the BM modified lotus leaf powder. For AT modification, 250 g of lotus leaf powder was mixed with 50 mL of distilled water. The mixture was subjected to autoclaving in the LDZX-5DKBS autoclave (Shanghai Shen An Medical Instrument Factory, Shanghai, China) at 121 °C under a pressure of 0.10 MPa for 30 min, and subsequently freeze-dried to obtain AT modified lotus leaf powder.

For the preparation of the lotus leaf IDF, 100 g of modified lotus leaf powder was combined with 1500 mL of hydrochloric acid adjusted to a pH of 4.0, and then 2.50 g of cellulase was added. The mixture was agitated at 50 °C and 250 rpm for 3 h, followed by centrifugation at 5000 rpm for 20 min to collect the precipitate. The precipitate was freeze-dried to obtain IDF. The three types of modified lotus leaf powder were processed to obtain SE modification of lotus leaf IDF (SEIDF), BM modification of lotus leaf IDF (BMIDF), and AT modification of lotus leaf IDF (ATIDF), respectively. In our previous study, relevant indicators were measured [13]. The crystallinity index, D_50_, and specific surface areas of SEIDF were 15.71%, 171.20 µm, and 74.47 m^2^/kg, respectively. The crystallinity index, D_50_, and specific surface areas of BMIDF were 19.31%, 15.55 µm, and 287.67 m^2^/kg, respectively. The crystallinity index, D_50_, and specific surface areas of ATIDF were 16.54%, 161.00 µm, and 61.17 m^2^/kg, respectively.

### 2.3. In Vitro Gastrointestinal Digestion

During different stages of in vitro gastrointestinal digestion, digestive juices of each sample were collected for the determination of total phenolic content (TPC), flavonoid composition, and antioxidant capacity. Thus, the release of polyphenols in the gastrointestinal digestion can be compared among SEIDF, BMIDF, and ATIDF. After in vitro gastrointestinal digestion, the IDF residues were utilized for in vitro colonic fermentation to assess the effects of SEIDF, BMIDF, and ATIDF on gut microbiota. In addition, the bound polyphenols cannot be released during gastrointestinal digestion but can be utilized in colonic fermentation. Therefore, the bound polyphenols of these IDF residues were also extracted, and TPC and antioxidant activity were determined.

The procedure for in vitro gastrointestinal digestion was assessed using the methodologies outlined in our previous study [19]. According to Table 1, simulated saliva fluid (SSF), simulated gastric fluid (SGF), and simulated small intestine fluid (SIF) were prepared.

For the simulated oral phase, samples (2 g) were mixed with 4 mL of SSF, 0.025 mL of 0.3 M CaCl_2_ solution, and 0.75 mL of 750 U/mL α-amylase solution. Then, supplemented with ultra-pure water to 10 mL, and shaken at 120 rpm, 37 °C for 5 min.

For the simulated gastric phase, digestive fluid was mixed with 8 mL of SGF. Then, 0.005 mL of 0.3 M CaCl_2_ solution, 0.667 mL of 300 U/mL pepsin solution, and 0.48 mL of 60 U/mL gastric lipase solution were added. The pH was adjusted to 3.0 using 6 M HCl and then supplemented with ultra-pure water to 20 mL. The mixture was shaken at 120 rpm, 37 °C for 3 h. During this period, samples were collected every hour (0, 1, 2, and 3 h) and centrifuged at −4 °C, 12,000 rpm for 15 min to obtain supernatants labeled as GP-0, GP-1, GP-2, and GP-3, respectively. These supernatants were stored at −80 °C for later determination of polyphenols and antioxidant activity.

For the simulated intestinal phase, digestive fluid was mixed with 8 mL of SIF. Then, 0.04 mL of 0.3 M CaCl_2_ solution, 3 mL of 204 U/mL trypsin solution, and 3 mL of 10 mM bile salt solution were added. The pH was adjusted to 7.0 with 5 M NaOH and then supplemented with ultra-pure water to 40 mL. Next, the mixture was shaken at 120 rpm, 37 °C for 3 h. During this period, samples were collected every hour (0, 1, 2, and 3 h) and centrifuged at −4 °C, 12,000 rpm for 15 min to obtain supernatants labeled as IP-0, IP-1, IP-2, and IP-3, respectively. These supernatants were stored at −80 °C for TPC, flavonoids composition, and antioxidant capacity determinations. The centrifuged residues after IP-3 were vacuum freeze-dried and stored at −80 °C for bound polyphenols and in vitro colonic fermentation.

### 2.4. Extraction of Bound Polyphenols

Bound polyphenol extraction of the remaining residue after in vitro gastrointestinal digestion was carried out as described by Dong et al. [20], with minor adjustments. The remaining residue was mixed with 10 mL of 2 M NaOH solution and shaken at 250 rpm for 3 h. The pH of the mixture was then adjusted to 2 using 6 M HCl, followed by centrifugation at 5000 rpm for 10 min. The supernatant was subjected to extraction with ethyl acetate, which was repeated three times. The collected ethyl acetate phase underwent solvent evaporation via vacuum rotary evaporation at 40 °C. Finally, the bound polyphenols were re-dissolved in distilled water for the determination of TPC and antioxidant activities.

### 2.5. Determination of TPC and Flavonoid Composition

TPC in digestive fluid and TPC of bound polyphenol extraction were determined with reference to our previous study methods [13], and the TPC was expressed as mg of gallic acid (GA) equivalent in per gram of sample (mg GA eq/g). During in vitro gastrointestinal digestion, flavonoid composition in digestive fluids was determined by HPLC, referring to the method in [13]. The standards analyzed included rutin, hyperoside, isoquercitrin, astragalin, and quercetin.

### 2.6. Determination of Antioxidant Capacity

The DPPH and ABTS free radical scavenging capacities were assessed using the methodologies outlined in our previous study [13]. Briefly, 0.1 mL of the sample was combined with 3.5 mL of 0.1 mmol/L DPPH and left to stand in darkness for 25 min. Then, the absorbance was measured at 517 nm using the UV-1780 Visible spectrophotometer (Suzhou Shimadzu Instruments Co., Ltd., Suzhou, China). The ABTS reserve solution was diluted with deionized water until it reached an absorbance of 0.70 ± 0.02 at 734 nm. After that, 0.05 mL of the sample was combined with 4.9 mL of ABTS solution and left to stand in darkness at room temperature for 10 min. Then, the absorbance was measured at 734 nm using the UV-1780 Visible Spectrophotometer. The results were presented as micromoles of Trolox equivalent per gram of the sample’s dry weight (µmol Trolox eq/g).

### 2.7. In Vitro Colonic Fermentation

The method for in vitro colonic fermentation was adjusted on the study by Liu et al. [21]. Fecal samples were obtained from two healthy individuals who had not received any antibiotic treatment in the past three months. The feces mixture (5 g) was mixed with 50 mL of sterile buffer solution to produce a 10% fecal slurry suspension. The medium (pH7) was prepared following the procedure described by Amorim et al. [22]. The freeze-dried residue (100 mg) was mixed with 9 mL medium, and then sterilized at 121 °C for 20 min. After cooling, 1 mL of fecal slurry suspension was added. The mixture was placed in a C-11 Mitsubishi anaerobic gas cylinder, with C-22 used as an indicator for anaerobic conditions, and incubated at 37 °C for 48 h. Following fermentation, the mixture was centrifuged at 4 °C, 12,000 rpm for 10 min. The supernatant was stored at −80 °C for the determination of short-chain fatty acids (SCFAs). The sediment was stored at −80 °C for the determinations of gut microbiota.

### 2.8. SCFA Analysis

SCFA analysis was carried out with reference to our previous study method [19], using the 1260 liquid chromatography system with high performance with the XB-C18 column (Agilent Technologies Co., Ltd., Santa Clara, CA, USA). Briefly, the supernatant after in vitro colonic fermentation was passed through a 0.45 µm membrane. High-performance liquid chromatography was conducted with the column temperature at 30 °C and a detection wavelength at 210 nm. The injection volume was 10 µL and the flow rate was 1.0 mL/min. The mobile phases consisted of a solution containing 95% phosphoric acid and 5% methanol.

### 2.9. Gut Microbiota Analysis

Gut microbiota analysis was carried out with reference to our previous study methods [19]. Briefly, the total genomic DNA of microorganisms was extracted from the sediment following in vitro colonic fermentation using the E.Z.N.A.^®^ soil DNA Kit (Omega Bio-tek, Norcross, GA, USA). The primer pairs 338F and 806R were utilized to amplify the V3–V4 hypervariable region of the bacterial 16S rRNA gene. The amplified products were then subjected to sequencing using an Illumina MiSeq PE300 platform (Illumina, San Diego, CA, USA).

### 2.10. Statistical Analysis

Data are expressed as the means ± standard deviation. The analysis of all samples was conducted in three separate trials. The comparison of multiple groups was conducted using ANOVA and Duncan multiple tests in IBM SPSS version 23. The threshold for statistical significance was set at *p* < 0.05. In gut microbiota statistical analysis, primary coordinate analysis (PCoA) is a multivariate statistical technique utilized to transform high-dimensional microbiome data into two- or three-dimensional coordinates, facilitating the observation of distinctions and similarities among samples. This study employed the unweighted-UniFrac distance algorithm to generate a two-dimensional PCoA figure. PC1 is the biggest principal component obtained through PCoA dimension reduction. The principal component, PC1, is obtained by calculating the covariance matrix of the original data, solving for its eigenvalues and eigenvectors, and selecting the eigenvector with the largest eigenvalue as the principal component. According to the PC1 value, the distribution dispersion of different groups of samples on the PC1 axis was represented by a boxplot. Linear discriminant analysis effect size (LEfSe) can be used to test differences in multiple levels of gut microbiota in order to identify communities that exhibit significant differences between samples. Linear discriminant analysis (LDA) is used to assess the impact of each marker on differences between groups. The calculation of LDA score specifically includes fitting the LDA model, obtaining the first feature vector, standardizing the first feature vector, calculating sample new coordinates, and calculating between-group distances based on grouping information as effect coefficients. Finally, the LDA score equals the effect coefficient multiplied by the standardized feature vector and then logarithmically transformed. This study used LDA > 2 and *p* < 0.05 to estimate the influence of each biomarker.

## 3. Results and Discussion

### 3.1. TPC During In Vitro Gastrointestinal Digestion

**Figure 1:**
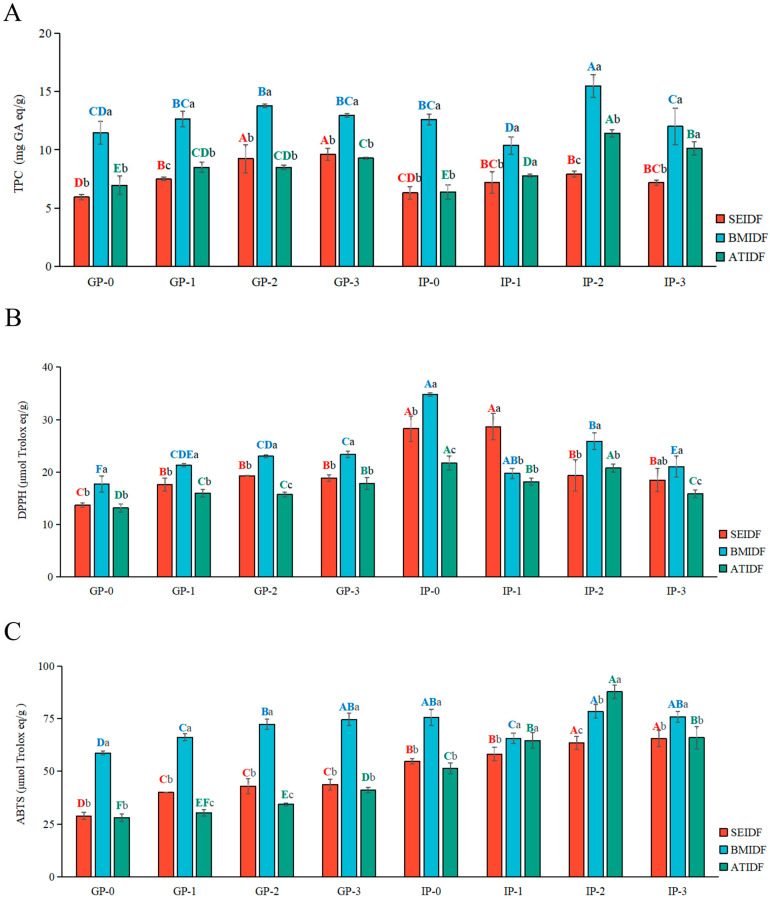
The release of TPC from SEIDF, BMIDF, and ATIDF (**A**), DPPH radical scavenging capacity (**B**), and ABTS radical scavenging capacity (**C**) of digestive fluids during in vitro gastrointestinal digestion. Capital letters (A–E) expressed significant differences of the same sample between different stages during in vitro gastrointestinal digestion (*p* < 0.05), lower case letters (a–c) indicate significant differences between the three IDFs at the same digested phase (*p* < 0.05). TPC, total phenolic content.

During the gastric digestion stage (GP-0 to GP-3), there was an overall increasing trend in the release of TPCs from SEIDF, BMIDF, and ATIDF, as shown in Figure 1A. The TPC released from BMIDF slightly decreased in the GP-2 to GP-3 stage. At the GP-3 stage, the TPCs released from BMIDF, SEIDF, and ATIDF were 9.60, 12.97, and 9.29 mg GA eq/g, respectively, which were 1.62, 1.13, and 1.34 times higher than that released at the GP-0 stage. This may be because the strong acid environment stimulates the hydrolysis of polyphenols and the conversion of some glycosides. Lucas-Gonzalez et al. [23] also observed a notable rise in TPC release from persimmon fruits during the gastric phase. In the initial stage of intestinal digestion (IP-0), the TPCs were reduced, possibly due to dilution by the addition of SIF, and alkaline pH and bile salts led to a decrease in polyphenols [24]. Subsequently, there was an increase in TPCs from the IP-1 to IP-2 stage, this may be because the intestinal digestive juices promoted the release of polyphenols [25]. The maximum polyphenolic contents during intestinal digestion were observed at the IP-2 stage, with contents of 7.93, 15.49, and 11.42 mg GA eq/g for SEIDF, BMIDF, and ATIDF, respectively. From the IP-2 stage to the IP-3 stage, there was a decrease in TPC which may be attributed to the poor stability of polyphenols during intestinal digestion. Overall, the three IDFs showed a similar pattern. The polyphenols released from SEIDF, BMIDF, and ATIDF increased in the gastric digestion stage, and increased first and then decreased in the intestinal digestion stage. Additionally, the TPC released from BMIDF was higher than that of SEIDF and ATIDF during in vitro gastrointestinal digestion, and TPC released from the GP-0 stage was significantly different from that at the IP-2 stage (*p* < 0.05).

BM technology is commonly employed for the purpose of reducing the particle size of raw materials and producing ultrafine powder [16]. In the planetary ball mill, lotus leaf powder was mixed with many small zirconia balls and then placed into the mill cylinder. During operation, the mill cylinder rotated and revolved simultaneously. The zirconia balls subjected the lotus leaf powder to high-intensity collision and grinding, resulting in a sharp decrease in particle size of the lotus leaf powder. According to our previous study [13], the D_50_ of BMIDF was 15.55 μm, which was lower than that of SEIDF (171.20 μm) and ATIDF (161.00 μm) (*p* < 0.05). In addition, the specific surface area of BMIDF was 287.67 m^2^/kg, which was higher than that of SEIDF (74.47 m^2^/kg) and ATIDF (61.17 m^2^/kg) (*p* < 0.05). Similar to the findings with Zhao et al. [8], the reduction in particle size and the increase in specific surface area can improve the interaction between BMIDF and digestive fluid, leading to the enhanced release of physical entrainment or non-covalently bound polyphenols. The high-temperature and high-pressure in AT modification did not significantly reduce the particle size of lotus leaf powder, and the high temperature may lead to enzymatic oxidation and non-enzymatic oxidation of polyphenols [26]. These factors may result in the lower release of polyphenols from ATIDF during gastrointestinal digestion. Escobedo et al. also showed that the AT modification reduces the polyphenol content in black beans [27]. Therefore, AT may contribute to the relaxation of fiber structure; however this method is not friendly to the functional components that are susceptible to heat. Different from the AT and BM modifications, the SE modification process takes place in a water solution, where the high-speed shearing of the fluid can cause loss of polyphenols in lotus leaf powder. This resulted in a lower release of polyphenols from SEIDF in gastrointestinal digestion compared to BMIDF. Overall, compared with SE and AT, the BM modification was more conducive to the release of polyphenols from lotus leaf IDF in gastrointestinal digestion.

### 3.2. Flavonoid Composition During In Vitro Gastrointestinal Digestion

Flavonoids are the functional active components of lotus leaves [28]. The flavonoids in lotus leaves mainly include rutin, quercetin, hyperoside, catechin, astragalus glycoside, isoquercitrin, and sutaxutein [12]. The chemical composition and content of flavonoids are related to the variety, growth condition, and geographical location of lotus leaves. In our previous study [13], five flavonoid components were identified from lotus leaf IDFs, which was similar to the results of Liao et al. [29]. In this study, isoquercitrin and astragalin were detected in SEIDF; hyperoside, isoquercitrin, astragalin, and quercetin were detected in BMIDF; rutin, hyperoside, isoquercitrin, and astragalin were detected in ATIDF. The content of the five flavonoids released from SEIDF, BMIDF, and ATIDF during in vitro gastrointestinal digestion is shown in Table 2.

During the gastric digestion stage, the isoquercitrin released from SEIDF and hyperoside, isoquercitrin, astragalin, and quercetin released from BMDIF showed a similar trend overall. They increased first, reached their highest value at the GP-2 stage, and then slightly decreased at the GP-3 stage. SEIDF was found to release low content of isoquercitrin. BMIDF was found to release hyperoside, isoquercitrin, astragalin, and quercetin, in which the contents of hyperoside and isoquercitrin at the GP-2 stage was 519.18 and 498.69 mg/100 g, respectively. During the GP-0 to GP-3 stage, ATIDF was found to release rutin, hyperoside, isoquercitrin, and astragalin. The four flavonoid contents increased continuously, and the contents of hyperoside and isoquercitrin at the GP-3 stage was 375.80 and 325.02 mg/100 g, respectively. During the intestinal digestion stage, almost none of the five flavonoids released from lotus leaf IDFs were detected. Only low levels of hyperoside, isoquercitrin, and astragalin were released by ATIDF at the IP-1 stage. Similar to the TPCs, flavonoids were diluted by intestinal digestive fluid, and alkaline conditions may lead to the degradation of the flavonoids [30]. Overall, BMIDF released more flavonoids than ATIDF during the gastrointestinal digestion. Among the three modifications, SE was the only one carried out in an aqueous solution [18]. Therefore, the SE modification process may lead to the loss of releasable polyphenols of lotus leaf powder, and thus the release of flavonoids in SEIDF was significantly lower than that in BMIDF and ATIDF during in vitro gastrointestinal digestion.

### 3.3. Antioxidant Capacity During In Vitro Gastrointestinal Digestion

Studies have shown that modification can lead to changes in the fiber structure and bioactive components of IDF [10], which may affect the antioxidant capacity of IDF during gastrointestinal digestion. During in vitro gastrointestinal digestion, the DPPH and ABTS radical scavenging capacities of SEIDF, BMIDF, and ATIDF are shown in Figure 1B and Figure 1C, respectively. The DPPH and ABTS radical scavenging capacities of the three IDFs all increased over time. The DPPH radical scavenging capacity of SEIDF, BMIDF, and ATIDF at the GP-3 stage were 1.38, 1.32, and 1.36 times higher than that at the GP-0 stage, respectively. The ABTS radical scavenging capacity of SEIDF, BMIDF, and ATIDF at the GP-3 stage were 1.51, 1.27, and 1.46 times higher than that at the GP-0 stage, respectively. Additionally, the DPPH and ABTS radical scavenging capacities of BMIDF were significantly higher than those of ATIDF and SEIDF (*p* < 0.05). The DPPH radical scavenging capacity of BMIDF was 1.31 and 1.24 times higher than that of ATIDF and SEIDF at the GP-3 stage, respectively. The ABTS radical scavenging capacity of BMIDF was 1.82 and 1.71 times higher than that of ATIDF and SEIDF at the GP-3 stage. Compared with the GP-3 stage, the DPPH and ABTS radical scavenging capacities of the three IDFs was slightly increased at the IP-0 stage. These results were different from the TPCs, which may indicate that the IDF of lotus leaves releases other antioxidant substances besides polyphenols during intestinal digestion. During the stage of intestinal digestion (IP-1 to IP-3), the DPPH and ABTS radical scavenging capacities of BMIDF and ATIDF showed the same trend as the gastrointestinal digestion stage, the DPPH radical scavenging capacity of SEIDF increased, and the ABTS radical scavenging capacity of SEIDF decreased. At the IP-3 stage, the DPPH radical scavenging capacity of BMIDF was 1.32 times that of ATIDF and 1.13 times that of SEIDF, and the ABTS radical scavenging capacity of BMIDF was 1.15 times higher than that of ATIDF and 1.16 times higher than that of SEIDF. Overall, the DPPH and ABTS radical scavenging capacities of BMIDF were higher than that of ATIDF and SEIDF during the in vitro gastrointestinal digestion stage, which was consistent with the results of TPCs.

### 3.4. TPC and Antioxidant Capacity of Bound Polyphenols After In Vitro Gastrointestinal Digestion

In plants, polyphenols are linked to DF through chemical bonds or physical entrainment. Among them, polyphenols are linked to DF by chemical bonds, such as hydrogen bonds, hydrophobic interactions, and covalent bonds. Some bound polyphenols are difficult to digest by the gastrointestinal tract and are eventually transported to the colon, where they may be released during the fermentation of gut microbiota, thereby affecting the gut microbiota composition and intestinal ecological environment [6,31]. It has been documented that the bioaccessibility of bound polyphenols during colonic fermentation is several times higher than that of the gastrointestinal digestive stage [6]. TPC and antioxidant capacity of bound polyphenols of IDF residues after in vitro gastrointestinal digestion are shown in Table 3. After in vitro gastrointestinal digestion, BMIDF showed the highest TPC of bound polyphenols, which was 2.03 times higher than that of SEIDF and 9.86 times higher than that of ATIDF, respectively. The DPPH and ABTS radical scavenging capacities of BMIDF and SEIDF bound polyphenols were significantly higher than that of ATIDF (*p* < 0.05). The DPPH radical scavenging capacity of BMIDF and SEIDF bound polyphenols was 1.58 and 1.48 times higher than that of ATIDF, respectively. The ABTS radical scavenging capacity of BMIDF and SEIDF bound polyphenols was 3.23 and 2.88 times higher than that of ATIDF, respectively. The ABTS and DPPH radical scavenging capacity of BMIDF were slightly higher than that of SEIDF. The results were consistent with the TPCs of SEIDF, BMIDF, and ATIDF in our previous study [13]. Overall, after in vitro gastrointestinal digestion, TPC of BMIDF residue bound polyphenols and antioxidant capacities were the highest. SEIDF had slightly lower levels than BMIDF, but both were significantly higher than those of ATIDF (*p* < 0.05). The results indicated that BMIDF and SEIDF may release more polyphenols during in vitro colonic fermentation and thus may have substantial regulatory impact on gut microbiota compared with ATIDF.

### 3.5. Gut Microbiota During In Vitro Colonic Fermentation

**Figure 2:**
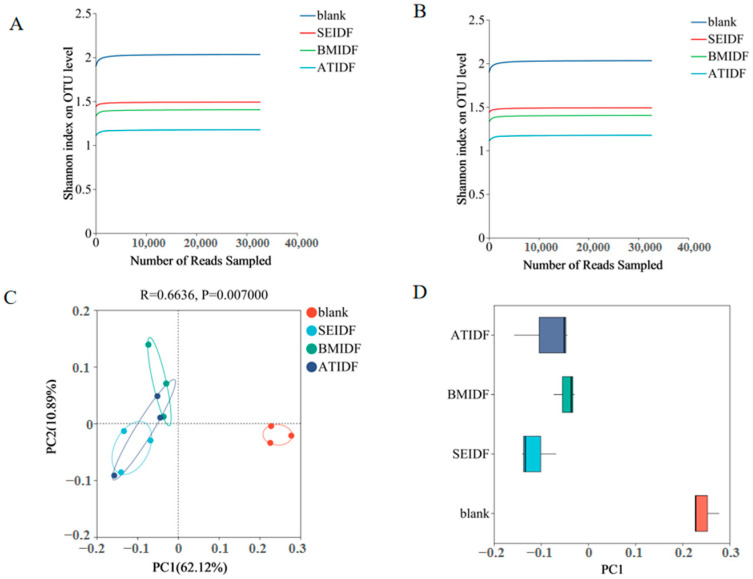
Shannon curves of gut microbiota (**A**), Sobs curves of gut microbiota (**B**), PCoA of gut microbiota (**C**), PC1 of gut microbiota (**D**). PCoA, primary coordinate analysis.

Colonic fermentation has become a convenient approach for examining the effect of undigested polysaccharides on gut microbiota [32]. The effects of SE, BM, and AT on the regulation of gut microbiota by lotus leaf IDFs were compared after in vitro colonic fermentation for 48 h. As shown in Figure 2A,B, the Sobs index and Shannon index curves exhibited a flattening trend as the number of sampled reads increased. The results suggested that the depth of sequencing and amount of data collected were adequate for accurately representing the richness and diversity of gut microbiota. In Figure 2C, the PCoA showed that PC1 and PC2 explained 62.12% and 10.89% of the variation in gut microbiota across all substrates, respectively. The blank group exhibited independence, whereas the lotus leaf IDF groups formed a cluster. The discrete distribution on PC1 (Figure 2D) also showed that there was a statistical separation between the blank group and the lotus leaf IDF groups, indicating low similarity between them. The results showed that lotus leaf IDFs changed the microbial species during in vitro colonic fermentation similar to the Cantu-Jungles study on fruit IDFs [33].

**Figure 3:**
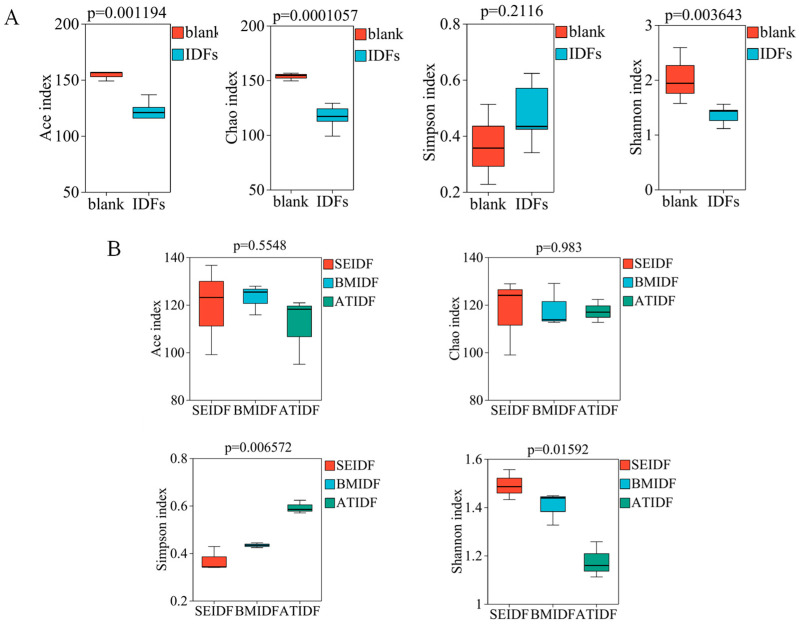
Alpha diversity of gut microbiota between the blank and lotus leaf IDF groups (**A**) and among the three lotus leaf IDF groups (**B**).

The results of alpha diversity of the blank group and the lotus leaf IDF groups are shown in Figure 3A. Ace and Chao are indicators of microbial community richness. Shannon and Simpson reflect gut microbiota species diversity. A higher Shannon value or a lower Simpson value indicates a greater diversity of gut microbiota. The Simpson index value in the lotus leaf IDF groups was slightly higher, while the Chao, Ace, and Shannon index values showed a significant decrease in the lotus leaf IDF groups compared to the control group. This result suggested the lotus leaf IDF groups reduced the richness and diversity of gut microbiota, which was consistent with previous studies that most IDFs are less fermentable or non-fermentable in the colon [9]. The differences between ATIDF, SEIDF, and ATIDF were further compared (Figure 3B). Among them, the Ace index of BMIDF and the Chao index of SEIDF were relatively high, but there is no significant difference. The Shannon index value of SEIDF was found to be significantly higher than that of BMIDF and ATIDF (*p* < 0.05), and the Simpson index value of SEIDF was significantly lower than that of BMIDF and ATIDF (*p* < 0.05). In general, there were no notable variations in the richness of gut microbiota among the three IDFs. Compared with BM and AT, SE modification of lotus leaf IDFs was more beneficial to the diversity of gut microbiota.

**Figure 4:**
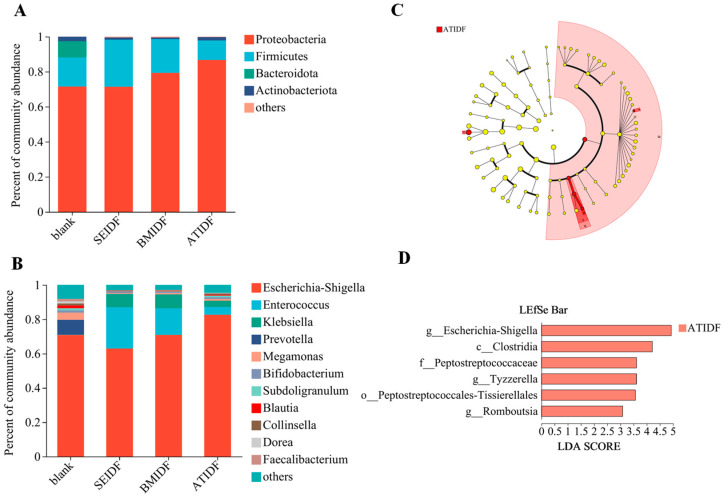
The relative abundance of gut microbiota at phylum (**A**) and genus (**B**) levels. The LEfSe of linear discriminant analysis among SEIDF, BMIDF, and ATIDF (**C**), the linear discriminant analysis (LDA score > 2, *p* < 0.05) (**D**).

As shown in Figure 4A, the blank group mainly consisted of Proteobacteria, Firmicutes, Bacteroides, and Actinomycetes. The three lotus leaf IDF groups were mainly composed of Proteobacteria, Firmicutes, and Actinomycetes. Proteobacteria is one of the most abundant phyla in human gut microbiota, which has been regarded as a characteristic of dysbiosis in gut microbiota [34]. Proteobacteria was the largest phylum of gut microbiota in this study. Compared with the relative abundance of Proteobacteria in the blank group (71.35%), BMIDF and ATIDF increased the abundance of Proteobacteria (79.28% and 86.64%). Firmicutes is the main phylum of healthy human gut microbiota. Some DFs can induce the growth of Firmicutes, the metabolites which have beneficial effects on human health [35]. In vitro colonic fermentation showed that the relative abundance of Firmicutes in the blank group, SEIDF, BMIDF, and ATIDF was 16.70%, 26.96%, 19.25%, and 11.13%, respectively. The lotus leaf IDFs modified by SE and BM increased the relative abundance of Firmicutes, while IDFs modified by AT decreased the relative abundance of Firmicutes. Bacteroides within the gut microbiota predominantly metabolize polysaccharides and oligosaccharides, thereby supplying essential nutrients and vitamins to both the human body and other microbial inhabitants of the intestinal tract [36,37]. The biodegradable polysaccharides and SDFs were found to be more conducive to the enhancement of Bacteroides abundance in gut microbiota. The relative abundance of Bacteroides was 9.35% in the blank group, while almost undetectable in the IDF groups, which may be related to the low content of degradable polysaccharides in lotus leaf IDFs. This result was consistent with Tian’s study, which observed a reduction in the abundance of Bacteroides and an alleviation of colitis in mice fed rice hull IDF [38]. The relative abundance of Actinomycetes was lower compared to Proteobacteria, Firmicutes, and Bacteroides. The relative abundance of Actinomycetes in the blank group (2.55%) was found to be greater than that in the IDF groups (0.97–1.92%). The result showed that lotus leaf IDFs modified by SE, BM, and AT reduced the relative abundance of Actinomycetes.

As shown in Figure 4B, Escherichia-Shigella, Enterococcus, Klebsiella, Prevotella, Megamonas, Bifidobacterium, Subdoligranulum, and Collinsella were dominant in all samples. Escherichia-Shigella is considered as a conditioned pathogen in the gut. Dysbiosis, caused by the significant expansion of Escherichia-Shigella, has been linked to the development of many diseases [39]. The relative abundance of Escherichia-Shigella in the blank group, SEIDF, BMIDF, and ATIDF was 70.63%, 62.87%, 70.84%, and 82.46%, respectively. The lotus leaf IDFs modified by SE decreased the relative abundance of Escherichia-Shigella, while the IDFs modified by BM and AT increased the relative abundance of Escherichia-Shigella. The relative abundance of Enterococcus and Klebsiella in the lotus leaf IDF groups showed a significant increase compared to the control group. The Enterococcus genus, a significant member of the Firmicutes phylum, is recognized as probiotics in both humans and animals [40]. In vitro colonic fermentation showed that lotus leaf IDFs modified by SE, BM, and AT increased the relative abundance of Enterococcus compared with the blank group. Among them, SEIDF had the best effect, followed by BMIDF. BMIDF showed the highest abundance of Klebsiella, followed by SEIDF. In addition, the relative abundance of Prevotella, Megamonas, Bifidobacterium, Subdoligranulum, Blautia, and Collinsella was reduced by SE, BM, and AT-modified lotus leaf IDFs. Among them, the relative abundance of Bifidobacterium, Megamonas, Subdoligranulum, and Collinsella in SEIDF and BMIDF was lower than that in ATIDF. Studies have confirmed that an increase in the relative abundance of Megamonas in gut microbiota was significantly associated with the progression of mild cognitive impairment [41], and Subdoligranulum triggered autoantibody-mediated transferable arthritis in gnotobiotic mouse models [42]. The modified lotus leaf IDFs reduced the relative abundance of Collinsella, from 1.16% in the blank group to 0.29% in SEIDF, 0.23% in BMIDF, and 0.77% in ATIDF. The Gomez-Arango et al. study showed that the relative abundance of Collinsella was negatively correlated with DF intake [43], which is consistent with our result. This result indicated that the intestinal fermentation capacity of SEIDF and BMIDF may be higher than that of ATIDF. The relative abundance of Prevotella was 1.25% in the blank group; however it was hardly detected in modified lotus leaf IDFs. The modified lotus leaf IDFs reduced the relative abundance of Bifidobacterium from 1.25% in the blank group to 0.90% in SEIDF, 0.69% in BMIDF, and 1.02% in ATIDF.

The LEfSe analysis among SEIDF, BMIDF, and ATIDF is shown in Figure 4C, and each biomarker is shown in Figure 4D. Among the three IDFs, six significant differences of OTUs were all in ATIDF. This result indicated that the regulatory effects of ATIDF on gut microbiota were significantly different from those of SEIDF and BMIDF. The biomarkers of ATIDF were Clostridia at the class level, Peptostreptococcales-Tissierellales at the order level, Peptostreptococcaceae at the family level, and Escherichia-Shigella and Tyzzerella-Romboutsia at the genus level. Among them, the relative abundances of Clostridia and Escherichia-Shigella exceeded 1%. Notably, the LDA of Escherichia-Shigella was the highest (4.926). The relative abundance of Escherichia-Shigella in ATIDF was 82.46%, which was significantly higher than that in SEIDF (62.87%) and BMIDF (70.84%). Li et al. demonstrated that fiber-bound polyphenols derived from highland barley exerted a significant inhibitory effect on the Escherichia-Shigella during in vitro colonic fermentation [44]. Hence, this result may be attributed to the significantly lower bound polyphenol content in ATIDF compared to SEIDF and BMIDF. Escherichia-Shigella, a conditioned pathogen in the gut, has been confirmed to be associated with various diseases, such as IgA nephropathy [39], inflammatory bowel disease [45], and mental disorders [46]. Clostridia belongs to the Firmicutes with a relative abundance of 5.19% in ATIDF, significantly higher than that in SEIDF (2.06%) and in BMIDF (2.60%). Clostridia is considered to be natural producers of ethanol and many higher alcohols, using lignocellulose and C1 gases as substrates [47]. The low crystallinity index of ATIDF may promote the interaction between Clostridia and lignocellulose substrates, leading to the proliferation of Clostridia. Moreover, Li et al. found that pomegranate polyphenols significantly inhibited the growth of Clostridia in fecal samples [48]. Therefore, the combined impact of the crystallinity index and bound polyphenols may have contributed to the higher relative abundance of Clostridia in ATIDF.

Overall, lotus leaf IDFs significantly affected the composition of gut microbiota. Among the three lotus leaf IDF groups, the IDF modified by SE was more beneficial for the diversity of gut microbiota, and exhibited lower relative abundances of Proteobacteria, Escherichia-Shigella, Megamonas, and Subdoligranulum, and higher relative abundances of Firmicutes and Enterococcus. BM modification had similar effects on regulating gut microbiota as SE modification. Compared with SE and BM modifications, AT modification increased the relative abundances of Proteobacteria and Escherichia-Shigella and decreased the relative abundance of Firmicutes. Thus, AT modification was less effective in regulating gut microbiota compared to SE and BM.

### 3.6. SCFA Concentrations After In Vitro Colonic Fermentation

**Figure 5:**
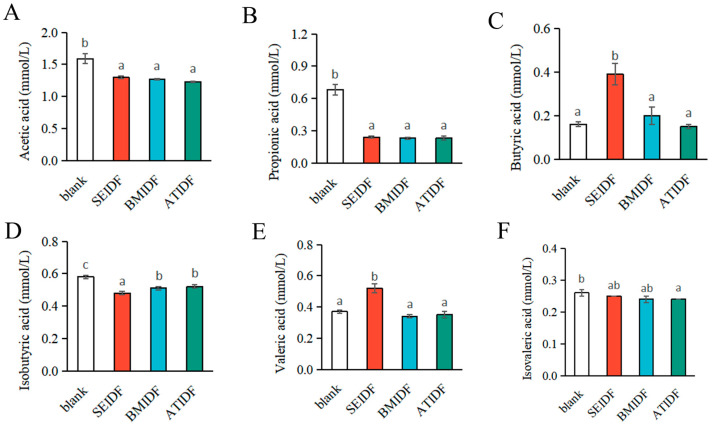
The concentrations of SCFAs, acetic acid (**A**), propionic acid (**B**), butyric acid (**C**), isobutyric acid (**D**), valeric acid (**E**), and isovaleric acid (**F**). Values with different letters were significantly different, *p* < 0.05.

SCFAs are the main metabolic products of DF fermentation by gut microbiota in the gastrointestinal tract and play a crucial role in maintaining the host’s health [49]. Studies have indicated that DFs produce SCFAs during colonic fermentation, mainly including acetic acid, propionic acid, and butyric acid [50]. The concentrations of SCFAs in the blank group, SEIDF, BMIDF, and ATIDF after 48 h of colonic fermentation are shown in Figure 5. Compared with the blank group, the concentrations of acetic acid, propionic acid, isobutyric acid in the IDF groups were significantly decreased (*p* < 0.05), and isovaleric acid was slightly reduced after 48 h of colonic fermentation. This result was consistent with previous studies that most IDFs are less fermentable or non-fermentable in the colon [9]. Notably, the concentrations of butyric acid and valeric acid in SEIDF were significantly higher than that in the blank group, BMIDF, and ATIDF (*p* < 0.05). Butyric acid can inhibit the active transport of bacteria, provide energy for colon cells, and prevent the occurrence of colon cancer by regulating apoptosis [51]. The concentration of butyric acid in SEIDF was 2.44, 1.95, and 2.60 times higher than that in the blank group, BMIDF, and ATIDF, respectively. Firmicutes is known for the significant role as a butyrate producer in the human gut [52]. Thus, the result may be attributed to the higher relative abundance of Firmicutes in SEIDF (26.96%) compared to the blank group (16.70%), BMIDF (19.25%), and ATIDF (11.13%). In addition, Zheng et al. showed that IDFs and bound polyphenols can promote the production of butyric acid in gut microbiota [53]. The bound polyphenols may also contribute to the increased concentration of butyric acid in SEIDF and BMIDF compared to the blank and ATIDF groups. Valeric acid exhibits anti-allergy activities through a variety of mechanisms, indicating its potential efficacy for addressing immune-mediated diseases [54]. The concentration of valeric acid in SEIDF was 1.41, 1.53, and 1.49 times higher than that in blank, BMIDF, and ATIDF groups, respectively. This may be related to the fact that Firmicutes is also one of the main producers of valeric acid [55]. Overall, compared to BM and AT modification, the SE modification of lotus leaves was more beneficial to produce SCFAs during in vitro colonic fermentation.

## 4. Conclusions

In this study, the effects of SE, BM, and AT on the in vitro gastrointestinal digestion and colonic fermentation characteristics of lotus leaf IDFs were compared. The results are consistent with those of our previous study on the physicochemical properties of lotus leaf IDFs using the three modified methods [13]. Among the three lotus leaf IDF groups, SEIDF was more beneficial for the diversity of gut microbiota, and significantly increased the concentrations of butyric acid and valeric acid during in vitro colonic fermentation. While BMIDF released the highest polyphenols and exhibited the strongest antioxidant capacity during in vitro gastrointestinal digestion. Compared to SEIDF and BMIDF, ATIDF showed almost no advantage in the release of polyphenols during in vitro gastrointestinal digestion and colonic fermentation characteristics. Further studies are needed to clarify the effects of SE, BM, and AT on the molecular structure of lotus leaf fibers, as well as the mechanism of regulating gut microbiota.

Overall, SE modification improved the colonic fermentation characteristics of lotus leaf IDFs more effectively, making SEIDF more suitable for the development of functional foods targeting gut microbiota regulation. While BM modification helped to promote the release of polyphenols from lotus leaf IDFs during in vitro gastrointestinal digestion, making BMIDF more suitable for the development of antioxidant functional foods. This study provides a theoretical basis for selecting a modification method for lotus leaf IDFs, and is conducive to the application of lotus leaf IDFs as a premium fiber additive in functional foods.

## Figures and Tables

**Table 1:** Electrolyte stock solutions of digestion fluid.

Input Salt Type	SSF (0.4 L)	SGF (0.4 L)	SIF (0.4 L)
KCl (5 M) (mL)	15.1	6.9	6.8
KH_2_PO_4_ (0.5 M) (mL)	3.7	0.9	0.8
NaHCO_4_ (1 M) (mL)	6.8	12.5	42.5
NaCl (2 M) (mL)	-	11.8	9.6
MgCl_2_(H_2_O)_6_ (0.15 M) (mL)	0.5	0.4	1.1
(NH_4_)_2_CO_4_ (0.5 M) (mL)	0.06	0.5	-
HCl (6 M) (mL)	0.09	1.3	0.7
CaCl_2_(H_2_O)_2_ (0.3 M) (mL)	0.025	0.005	0.04
	supplemented to 0.4 L with ultra-pure water		

**Table 2:** Flavonoid composition during in vitro gastrointestinal digestion. (mg/100 g).

Gastrointestinal Digestion Phase	Rutin	Hyperoside	Isoquercitrin	Astragalin	Quercetin
SEIDF					
GP-0	-	-	2.44 ± 0.06	-	-
GP-1	-	-	3.12 ± 0.28	-	-
GP-2	-	-	4.64 ± 0.34	-	-
GP-3	-	-	4.41 ± 0.83	-	-
IP-0	-	-	-	-	-
IP-1	-	-	-	-	-
IP-2	-	-	-	-	-
IP-3	-	-	-	-	-
BMIDF					
GP-0	-	388.39 ± 11.55	389.05 ± 17.11	50.26 ± 3.02	6.86 ± 0.23
GP-1	-	454.77 ± 24.48	442.20 ± 23.49	55.27 ± 2.29	21.34 ± 0.39
GP-2	-	519.18 ± 25.44	498.69 ± 21.76	62.78 ± 4.05	34.99 ± 8.88
GP-3	-	499.95 ± 5.01	489.01 ± 8.74	60.69 ± 1.62	26.10 ± 1.86
IP-0	-	-	-	-	-
IP-1	-	-	-	-	-
IP-2	-	-	-	-	-
IP-3	-	-	-	-	-
ATIDF					
GP-0	12.49 ± 0.46	229.61 ± 35.95	195.56 ± 30.13	29.79 ± 4.83	-
GP-1	15.49 ± 3.96	262.07 ± 89.79	223.50 ± 76.69	34.36 ± 11.96	-
GP-2	20.43 ± 7.03	348.57 ± 133.94	298.57 ± 113.18	45.83 ± 16.10	-
GP-3	21.31 ± 3.33	375.80 ± 54.28	325.02 ± 47.39	49.00 ± 7.23	-
IP-0	-	16.90 ± 16.82	13.77 ± 13.58	12.52 ± 5.99	-
IP-1	-	-	-	-	-
IP-2	-	-	-	-	-
IP-3	-	-	-	-	-

Values are presented as means ± sd (n = 3). “-” indicates non-detection.

**Table 3:** TPC and antioxidant capacity of bound polyphenols.

Group	SEIDF	BMIDF	ATIDF
TPC of bound polyphenols (mg GA eq/g)	3.21 ± 0.25 ^b^	6.51 ± 0.78 ^a^	0.66 ± 0.03 ^c^
DPPH radical scavenging capacity (μmol Trolox/g)	30.40 ± 2.25 ^a^	32.23 ± 2.43 ^a^	20.43 ± 1.04 ^b^
ABTS radical scavenging capacity (μmol Trolox/g)	29.87 ± 2.34 ^a^	33.51 ± 1.41 ^a^	10.38 ± 1.03 ^b^

Values are means ± sd (n = 3). Values with different letters were significantly different in same line, *p* < 0.05.

## Data Availability

The original contributions presented in the study are included in the article; further inquiries can be directed to the corresponding author.
